# Interprofessional Education Activity to Improve Communication With Older Adults With Hearing Loss

**DOI:** 10.1093/geroni/igaa057.011

**Published:** 2020-12-16

**Authors:** Laura Gaeta, Tara Sharpp

**Affiliations:** California State University, Sacramento, Sacramento, California, United States

## Abstract

The purpose of this study was to determine how an interprofessional education (IPE) intervention with Communication Sciences and Disorders (CSAD) and Nursing students can affect their ability to communicate effectively with older adults who have hearing loss. As the older adult demographic increases, healthcare professionals must provide competent care, which includes effectively managing hearing-related communication difficulties in an increasingly diverse population. Faculty received IRB approval to conduct a descriptive mixed-methods study to determine knowledge and satisfaction of students completing an IPE activity. Students were divided into teams of CSAD and Nursing students. Students listened to a brief presentation on IPE before they were introduced to a complex case study of an 84-year-old male with age-related hearing loss. We administered a knowledge assessment questionnaire (KAQ) we created regarding communication with older adults before and after the activity. A total of 92 participants in the two programs (n=36 CSAD, n=56 Nursing) completed the KAQ before and after the activity and an evaluation with a Likert-type scale and open-ended questions. CSAD students scored significantly higher than Nursing students on the KAQ at baseline (F=25.69, p<0.001) and KAQ scores increased significantly (F=57.04, p<0.001) among both groups from pretest to posttest. The evaluation data indicated students were able to learn other perspectives and found the experience valuable. Based on the improvement in scores on the KAQ and evaluation data, this IPE activity increased knowledge related to communication with older adults with hearing loss and awareness of the roles of other professions.

